# Implementation of weight-inclusive care in primary care: a mixed-methods pilot study of a brief, behavior-focused provider training

**DOI:** 10.3389/fpubh.2026.1934441

**Published:** 2026-09-11

**Authors:** Mindy L. McEntee, Rose Zach, Binoli Herath

**Affiliations:** 1 College of Health Solutions, Arizona State University, Phoenix, AZ, United States; 2 College of Medicine, University of Arizona, Phoenix, AZ, United States

**Keywords:** intervention, primary care, providers, weight stigma, weight-inclusive care

## Abstract

**Introduction:**

Weight stigma remains prevalent in health care, adversely impacting patient outcomes and perpetuating health disparities. Weight-inclusive care (WIC) aims to improve health independent of weight through personalized and sustainable changes to health promoting behaviors and reduced weight stigma, yet little research has examined how to support its implementation in primary care.

**Methods:**

This convergent parallel design mixed methods study piloted a brief pragmatic intervention to facilitate adoption of WIC at a federally qualified health center, evaluating feasibility, acceptability, and preliminary implementation outcomes focused on adoption of WIC clinical behaviors rather than attitude change. 10 providers participated in the training, with four completing follow-up interviews. Participants completed online surveys at baseline (*n* = 9) and two-week follow up (*n* = 10) assessing acceptability, attitudes, and self-reported clinical behaviors. Quantitative data were analyzed using Wilcoxon matched-pairs signed-rank tests, thematic analysis was used to analyze interview transcripts for further insight into the implementation of practice changes.

**Results:**

Although changes in self-reported attitudes and clinical behaviors were not statistically significant, providers reported making modifications to the physical environment and clinic workflows in qualitative interviews, including changes that affected weighing procedures, documentation, communication strategies, and improved accessibility of appropriately sized equipment. Interviews further identified organizational and structural barriers to implementation and revealed a recurring disconnect between WIC knowledge and application.

**Discussion:**

Findings suggest adoption of WIC involves more than acquiring new knowledge and may require fundamental changes to clinical reasoning, organizational support, and healthcare infrastructure. Behavior-focused implementation strategies may facilitate early adoption of WIC and represent a promising alternative to traditional attitude-focused interventions. Organizational support and accountability mechanisms are likely needed to effectively support this paradigm shift. Larger studies with validated behavioral measures and longer follow-up are needed to evaluate sustained implementation and patient outcomes.

## 1 Introduction

Modern medicine assumes higher weight is inherently unhealthy and treatable through weight loss. Providers using this weight-centric care (WCC) approach emphasize weight through the use of BMI screening, setting and monitoring weight-loss treatment goals, counseling patients to lose weight through calorie reduction, dietary restriction, and/or increased physical activity, and may prescribe medications or refer to bariatric surgery for individuals unable to lose weight through diet and exercise alone. Yet WCC has faced empirical, ideological, and technical critiques (1) for oversimplifying complex biological, socio-ecological, and environmental systems contributing to weight (2–4). Critically, research supporting this paradigm conflates correlation with causation and fails to distinguish weight from health behaviors or adjust for confounds including weight stigma, diet/weight history, stress, and social determinants of health (SDOH) (1, 5–9). Dieting studies suggest intentional weight loss is rarely substantial or sustained (10–16), often leading to weight cycling (17–19), weight stigma (20–23), and/or disordered eating (14, 24–26), all of which are associated with adverse effects attributed to higher weight (27–37).

Weight stigma includes labeling, stereotyping, separation, status loss, and discrimination based on body weight, shape, or size, affecting all ages (35). Stigma is a fundamental cause of population health inequities in healthcare, where it influences physiological, psychosocial, and behavioral processes, limits access/quality of protective resources, and contributes to adverse health outcomes (36). Adverse effects occur bidirectionally through multiple pathways, including elevated HbA1c, cortisol, oxidative stress, C-reactive protein, and other proinflammatory cytokines, decreased nutrient absorption, binge eating, decreased physical activity, disordered eating and/or exercising, social isolation, and healthcare avoidance, increasing morbidity and mortality (1, 32, 35, 37).

Within WCC, providers face a paradoxical call to reduce weight stigma while continuing to promote weight loss/management. For example, providers may be encouraged to adopt first-person language, use patient-preferred terminology, ask permission to discuss weight, and avoid making lifestyle assumptions while continuing to use BMI, setting or monitoring weight-loss goals, counseling patients on weight loss, and recommending weight-loss medications or bariatric surgery. Even when communicated respectfully, retaining weight loss as a primary treatment goal may unintentionally perpetuate weight stigma and contribute to feelings of shame or self-blame, internalized weight bias, reduced trust in providers, and avoidance of healthcare. Many studies similarly denounce weight stigma while continuing to use medical terminology referring to higher weight as a disease; use of terms such as “obesity” perpetuate stigma by pathologizing fatness, promoting inappropriate use of body mass index as an individual indicator of health, and equating weight loss with improved health (23, 38–40). Use of weight as a proxy for individual health is further problematic in that it overlooks evidence that behavior change can improve more direct health markers- including glucose tolerance, blood pressure, resting heart rate, and inflammatory markers- independent of weight loss (41–50), making this both a science and social justice issue.

From a social justice standpoint, WCC places disproportionate responsibility on individuals to change their bodies while overlooking social, economic, and structural factors that shape health. Weight stigma can also intersect with other forms of discrimination and contribute to inequitable healthcare experiences, particularly when providers attribute the concerns of patients in larger bodies to their weight at the expense of equitable assessment and care. Thus, addressing weight stigma requires more than changing provider language; it also requires examining the underlying assumptions and clinical practices that position WCC as necessary for health. Without addressing the underlying assumptions and behaviors perpetuating weight stigma, these problems are likely to persist.

Weight-inclusive care (WIC) promotes health improvement independent of weight, adhering to principles of beneficence and nonmaleficence through collaborative development of personalized, sustainable changes to health promoting behavior and intentional efforts to reduce stigma (48). Critically, while WCC promotes behavior change *in the service of weight loss*, WIC acknowledges health-promoting *behaviors* can improve clinical and psychological outcomes, regardless of weight change. Instead of monitoring weight, WIC treatment focuses on behavior change, improvements in wellbeing (e.g., increased energy, improved mood, reduced pain, etc.,) and other objective health markers such as blood pressure, lipids, hemoglobin A1C, cardiorespiratory fitness, and functional mobility. By separating health improvement from weight loss, WIC stands to increase equitable care, yet there remains little guidance on how to effectively implement this paradigm shift. This study therefore evaluated a brief intervention designed to help primary care providers (PCPs) adopt WIC practices, assessing training feasibility, acceptability, perceived relevance, and preliminary changes in provider attitudes and behaviors at two-week follow-up.

## 2 Theoretical rationale for a behavioral approach

In contrast to other weight stigma interventions, provider attitude change was not considered a necessary prerequisite for changed behavior in patient care. This approach was informed by radical behaviorism (51), which views behavior as being shaped by environmental antecedents and consequences and modern contextual behavioral science (52), which extends this functional perspective by emphasizing behavior in context rather than assuming changes in internal attitudes or beliefs must precede behavior change. As such, training targeted modifiable clinical practices and contextual factors that could directly cue, support, and reinforce WIC implementation, including workflows, assessment and case-formulation practices, clinical decision making and counseling practices, documentation policies, reinforcement of health behaviors over weight, and features of the clinical environment. In practical terms, training focused on what providers could say, ask, recommend, document, measure, reinforce, and do during their patient encounters to implement WIC. This behavior-focused approach is notable given attitude-change interventions (53, 54) have demonstrated limited efficacy and little research has focused directly on subsequent provider behavior.

## 3 Materials and methods

Authors adapted a structural competency intervention into a one-hour interactive presentation for PCPs based on current literature and behavior change principles. Training clarified terminology, reviewed problematic assumptions of WCC, and promoted WIC aligned patient-centered strategies distinguishing health from weight through development of individualized sustainable behavior change goals like increased fruit/vegetable intake (vs. restriction), enjoyable physical activity, stress management, and improved sleep quality. The intervention also addressed personal biases, assumptions underlying WCC, the importance of SDOH, fostering supportive environments, and collaboratively discussed case studies using WIC practices. Training was co-delivered by two presenters utilizing an interactive format that combined didactic instruction with case presentation, review, and facilitated discussion. Intentional efforts were made to foster psychological safety by establishing a respectful, non-judgmental learning environment that encouraged self-reflection and willingness to explore sensitive or challenging topics. Provider strategies to facilitate WIC adoption included reviewing evidence underlying medical decision-making, respecting patients' health priorities and chief complaint, broadening case formulation to include SDOH, respectful communication practices regarding weight and health, and accessing supplemental WIC resources.^1^ Training aimed to elicit ideas from participants as much as possible to increase relevance and engagement, with additional recommendations published by the Association for Size Diversity and Health (ASDAH) and the National Association to Advance Fat Acceptance (NAAFA) incorporated into the discussion as needed.

This convergent parallel mixed methods study evaluated feasibility and acceptability of the training along with preliminary reported changes in provider attitudes and behaviors. This study was approved by the Arizona State University Institutional Review Board and piloted at a federally qualified health center in Phoenix, Arizona.

Site selection was informed by practical considerations, including logistical feasibility and an established partnership between the study team and participating institution, including ongoing collaboration with research activities. Authors coordinated with FQHC leadership to arrange for training to occur during a standing provider meeting to promote attendance. Leadership also agreed to provide 15 min of clinic time for questionnaire completion to facilitate data collection (at both baseline and follow up). Participants completed online surveys using anonymous reproducible IDs to link responses. All attending providers completed the training; survey completion was encouraged but optional. Demographic information (age, race, ethnicity, gender) and clinician characteristics (years as licensed provider, number of patients seen per week) were collected at baseline, prior to training. Participants were sent up to three email reminders from leadership containing a link to complete the two-week follow-up survey and optional sign-up for participation in a 30-min zoom interview.

### 3.1 Training acceptability and perceived relevance

Participants rated the importance of research and weight bias training for clinical practice pre and post training. Post-training, they assessed their ability to distinguish between WCC and WIC, communicate respectfully with patients about weight and health, and work collaboratively with patients to set realistic, non-weight focused goals.

### 3.2 Provider attitudes and practice behaviors

Participant attitudes and clinical practice behaviors were evaluated using items adapted with permission from previous studies evaluating weight bias among PCPs (55), and eating disorder treatment professionals (56). Wording was modified to avoid stigmatizing terminology (2); additional behavioral items were added to reflect a range of WCC and WIC practices based on recommendations published by the Association for Size Diversity and Health (ASDAH) and the National Association to Advance Fat Acceptance (NAAFA). Providers were asked to rate their level of agreement with 12 attitudinal statements and how frequently they engaged in 23 clinical practices. All items were scored on five-point Likert scales with higher scores indicating greater ability/agreement/use.

### 3.3 Preliminary adoption and implementation of WIC

PCPs provided qualitative feedback on the training and noted planned practice changes on follow-up surveys. Participants could also opt-in to semi-structured follow-up interviews conducted via Zoom: [redacted initials] led the interviews, [redacted initials] recorded, transcribed, and anonymized responses. Providers described their experiences treating higher-weight patients and practice changes implemented post-intervention and discussed case conceptualization and treatment recommendations for a hypothetical patient (Supplementary File 1). After reading through a standardized patient vignette, participants were asked to walk the interviewers through their thought process as they considered (a) how they'd react and (b) what they'd recommend or do for this patient. All interview participants received $50 electronic gift card incentives.

### 3.4 Data analysis

Quantitative data was analyzed using IBM SPSS statistical software version 29.0. Descriptive statistics examined demographic and clinician characteristics. Wilcoxon tests evaluated changes in attitudes and practice behaviors due to non-normal data distribution. Interview transcripts were evaluated using deductive and inductive thematic analysis to explore provider experiences with WIC training, facilitators and barriers to implementation, and impact on patient assessment and treatment formulation using a standardized patient vignette. A preliminary codebook based on research examining causal attributions of body weight (57) was supplemented by an inductive approach to identify additional themes. All authors coded transcripts independently and resolved disagreements through discussion.

## 4 Results

Nine providers completed baseline surveys; post-intervention included an additional participant who attended training but did not complete the baseline survey (*n* = 10). Demographic data and years in practice are displayed in Table 1.

**Table 1:** Participant demographics and weight-stigma experience (*n* = 9).

Participant characteristics	*n*	*%*
Gender		
Male	1	11.1
Female	8	88.0
Age range		
22–34	2	22.2
35–44	3	33.3
45–54	1	11.1
55–64	3	33.3
Race/ethnicity		
Asian	1	11.1
Black	2	22.2
White	5	55.5
Years in practice		
1–5 years	4	44.4
5–10 years	2	22.2
10–15 years	1	11.1
More than 15 years	2	22.2
Personal history (*n* = 8)		
Currently trying to lose weight	5	63
Personal history of disordered eating or an eating disorder	1	13
Been teased due to own body weight, size, or shape	6	75
Treated unfairly due to own body weight, size, or shape	2	25
Experienced discrimination due to body weight, size, or shape	2	25

### 4.1 Training acceptability and perceived relevance

Overall feedback indicated training was well-received. All providers indicated research was at least moderately important to their clinical practice at baseline, with 63% (*n* = 5) rating research as “very” or “extremely” important. This rose to 90% post-intervention (*n* = 9), a statistically significant increase (*z* = 2.00, *p* = 0.046). All providers rated the importance of WIC training as at least moderately important to their clinical practice at baseline, with 50% indicating (*n* = 4) it was “very” or “extremely” important. This increased to 100% post-intervention (*n* = 10, *z* = 2.24, *p* = 0.025).

Post-training, providers agreed they were able to distinguish WCC from WIC in practice, communicate clearly and respectfully about weight and health, and work collaboratively with patients to set realistic, non-weight focused goals post-training (Supplementary Figure 1).

### 4.2 Provider attitudes and practice behaviors

Wilcoxon signed-rank tests indicated no statistically significant change in self-reported attitudes or clinical behaviors following training (*p* > 0.05). WCC data is presented in Table 2, WIC data in Table 3.

**Table 2:** Weight-centric attitudes and behaviors.

Weight-Centric Attitudes and Behaviors	Baseline (*n* = 9)	Post-intervention (*n* = 10)	Wilcoxon signed-rank test	
	Mean (SD)	Mean (SD)	*z*	*p*
*Weight-Centric Attitudes*				
Research is important to improving my clinical practice.	3.88 (0.84)	4.40 (0.70)	2.00	0.046
WIC training is/was important to improving my clinical practice.	3.63 (0.74)	4.20 (0.42)	2.24	0.025
I believe it's necessary to educate higher weight patients on the health risks associated with their weight.	4.44 (1.01)	3.70 (1.16)	−1.51	0.13
I feel uncomfortable when examining a larger bodied patient.	2.00 (1.23)	2.20 (1.14)	0.71	0.48
I have negative reactions toward the appearance of higher weight or larger bodied patients.	1.78 (0.83)	2.00 (1.25)	−0.14	0.89
I often feel frustrated with higher weight patients.	2.00 (1.12)	1.90 (1.20)	−0.37	0.71
I am usually successful in helping higher patients lose weight.	3.11 (1.17)	3.10 (0.74)	0	1.0
It is difficult for me to feel empathy for a heavier patient.	1.78 (0.83)	2.00 (1.33)	0	1.0
My peers tend to have negative attitudes toward higher weight patients.	2.67 (1.00)	2.90 (1.20)	0.71	0.48
I have heard/witnessed other providers making negative comments or jokes about higher weight patients.	2.89 (1.54)	2.80 (1.32)	−0.63	0.53
*Weight-Centric Behaviors*				
Document or review a patient's weight as a routine part of clinic workflow.	4.50 (0.54)	4.20 (0.63)	−1.34	0.18
Talk about food as fuel or medicine.	3.63 (1.06)	3.60 (0.97)	−0.33	0.74
Provide dietary recommendations (e.g. recommend a specific diet, give guidance on foods and/or supplements).	4.00 (0.76)	3.90 (0.99)	0.58	0.56
Explain that even modest weight loss can improve patient health outcomes.	4.38 (0.52)	4.30 (0.68)	0.00	1.0
Recommend evaluation by a surgeon if a patient meets appropriate criteria for weight loss surgery (BMI>40, significant comorbidities).	3.50 (1.20)	3.30 (1.25)	0.00	1.0
Verbally appreciate success in weight loss, even if weight loss is minimal.	4.63 (0.74)	3.90 (0.88)	−1.41	0.16

**Table 3:** Weight-inclusive care behaviors.

Weight-Inclusive Behaviors	Baseline (*n* = 9)	Post-intervention (*n* = 10)	Wilcoxon signed-rank test	
	Mean (SD)	Mean (SD)	*z*	*p*
Intentionally make accommodations for larger bodied patients, including use of appropriately sized blood pressure cuffs, examination gowns, and armless chairs.	4.13 (0.64)	3.90 (0.74)	−1.34	0.18
Respect the patient's health priorities and address their chief complaint.	4.75 (0.71)	4.40 (0.52)	−1.34	0.18
Avoid using certain terms to describe body weight that may o?end patients.	4.38 (1.06)	4.30 (0.68)	0.0	1.0
Ask for permission to discuss body weight and the patient's preferred language for doing so.	3.00 (1.41)	3.70 (0.82)	−1.41	0.16
Engage in health-centered, non-weight focused language.	3.75 (0.71)	4.40 (0.70)	−1.89	0.06
Provide information about the complex relationship between weight and health, weight controllability, and weight science.	3.63 (1.30)	3.60 (0.84)	−0.11	0.92
Listen to and try to understand the context surrounding the patient's concerns.	4.50 (0.54)	4.60 (0.52)	−1.0	0.32
Ask rather than make assumptions about their engagement in health promoting behaviors.	4.50 (0.76)	4.30 (0.68)	−1.0	0.32
Acknowledge food is part of culture and eating is a social and emotional experience.	4.00 (0.76)	3.90 (0.99)	−0.71	0.48
Verbally acknowledge patient improvements in health behaviors.	4.50 (0.76)	4.30 (0.48)	−1.0	0.32
Collaborate with the patient to set realistic behavioral goals related to health behavior change rather than weight.	4.13 (0.64)	4.20 (0.63)	−0.45	0.66
Counsel patients on exercise without linking it to weight.	4.25 (0.71)	4.0 (0.82)	−1.41	0.16
Reflect on your own biases and assumptions about the patient's weight and how it may impact the care you provide.	4.00 (0.76)	3.80 (0.92)	−0.82	0.41
Reflect on your own biases about healthism and the moralization of health behavior.	3.87 (0.64)	3.80 (0.92)	−0.38	0.71

### 4.3 Preliminary adoption and implementation of WIC

Practice changes reported on the two-week follow-up survey included establishing a more weight-inclusive clinical environment through increased availability of larger blood pressure cuffs, emphasizing healthy food additions over dietary restriction, prioritization of behavioral health, and use of new patient communication strategies. Provider feedback on the training noted appreciation for the discussion of weight loss medication risks, centering patients' experience, and identified a need for more accessible resources.

Four providers completed follow-up interviews. All interviewees reported implementing workflow changes for weighing patients: half no longer required weighing at each visit, the other half moved the scale to increase privacy. Two providers reported efforts to ensure appropriately sized equipment was more readily available. One interviewee increased their personal efforts to anticipate patient needs. The most commonly reported barrier to WIC adoption and implementation was limited resources, which encompassed a lack of appropriately sized equipment, difficulty rearranging clinic space and modifying workflows, being short-staffed and facing time pressure seeing clients, a need for WIC-consistent handouts, and knowledge of other WIC providers to refer to and consult with. Providers also suggested including other clinic staff in training, ongoing training sessions, monthly check-ins, establishing formal goals and plans for accountability, virtual shadowing to provide feedback, and clarification of clinic-wide weighing practices/documentation to improve WIC practice.

Review of the standardized patient vignette provided insight into each providers' understanding of the training material. Analysis categorized clinical formulation and treatment recommendations into three themes: weight-centric recommendations, weight-inclusive recommendations, and a disconnect in which providers conflated the two paradigms. The disconnect theme highlighted critical gaps between knowledge and clinical application of WIC principles.

Weight-centric care (WCC) equated weight with health and emerged 19 times. Some providers acknowledged complexity of weight while still using it as a proxy for health. As participant 1 noted,

“*There's a lot of factors that go into someone's health when they are at an unhealthy weight, such as high blood pressure, sleep apnea… we can potentially mitigate with someone being a healthier weight.”*

WCC appeared in both case formulation and treatment recommendations for the standardized patient vignette. As participant 1 explained,

“*If this was my patient, I understand her concern is her weight. But as a provider, I feel the highest concern is for osteoarthritis. By me managing her osteoarthritis, and therefore reducing her pain, she is then able to then hopefully exercise and do activities that can promote weight loss.”*

While osteoarthritis treatment was the provider's primary concern, the commentary suggests pain reduction aimed to facilitate weight loss. Participant 2 similarly endorsed weight loss:

“*I know that the weight is something that we will need to address, because it does look like it's impacting her life.”*

One participant discussed their treatment recommendations beyond the provided patient vignette. Participant 1 mentioned how they approach dietary choices:

“*I give them the explanation of, ‘You have a 2-liter soda. You have a bottle of soda. You have a canned soda, and you have a little itty bitty soda, you know – you can still have those pleasures, but choose the smallest of them.”'*

While likely intended to demonstrate the patient can enjoy their favorite foods in moderation, emphasis on portion control without further behavioral assessment or collaborative exploration of goals suggests an underlying goal of weight management, characteristic of WCC.

Weight-inclusive care (WIC) sought to improve health without targeting weight and emerged 21 times across interviews. Participant 2 reflected on her practice experience:

“*I feel like some of the patients in our population are maybe hesitant to seek care with us because we are not being as inclusive as we should be, and I think that's where that dread comes from.”*

Participant 3 incorporated WIC practices to evaluate contextual factors affecting food choice and promote intuitive eating in the case scenario:

“*I'd want to know how she's cooking. Does she have a mat that is cushioned? Is she standing on a hard floor, is she wearing shoes? Does she have a seat that she could potentially sit in while she's cooking or a table that she could prepare her food at? That makes it a little bit easier… because she's probably eating things that are not always as nutritious, or maybe not even what she wants to eat.”*

Participant 2 shared another WIC practice, further evaluating symptoms rather than automatically attributing them to weight:

“*Let's do some bloodwork. I always want to start there. Because what if it's not osteoarthritis? What if it is rheumatoid arthritis? What if there are some other things going on that are contributing to her symptoms?”*

The “disconnect” theme involved misinterpretation or misapplication of WIC, reflecting an underlying goal of weight loss/control to improve health. Early in the interview, participant 1 noted,

“*what their number is on the scale does not determine whether they're healthy or unhealthy….It's more than just the number on the scale that determines someone's health.”*

This participant went on to note,

“*What I find challenging for some patients… I'm providing them the medication that they need to manage their chronic conditions, whether it be obesity, diabetes, high blood pressure…. but they aren't making any changes with their lifestyle to hopefully get them off of these medications and lose weight to become healthier.”*

In these quotes, an initial attempt to distinguish weight from health is later followed by traditional weight centric care, endorsing weight loss to improve health. A total of 15 disconnects were observed during interviews.

Another example was observed in participant 1′s case scenario:

“*...see what resources are available for her being that she is a veteran. Whether it's physical therapy, whether it's getting her memberships into something like the Y.M.C.A. Or see what VA resources there are that can assist her with her overall health. Not in the sense of weight management, but to make her life easier where she can address her weight.”*

Although recommending additional health resources and insisting that weight management was not the purpose of referral, weight loss remained the end treatment goal.

Finally, participants identified several organizational factors that could affect WIC implementation. These included limited resources and equipment, competing organizational priorities, perceived limited support from leadership, lack of team cohesion, and prior training grounded in WCC. Participants also noted a need for ongoing support to facilitate implementation. These factors were raised less frequently and were not discussed in sufficient depth to constitute a distinct theme but provided additional context regarding potential challenges to implementation.

## 5 Discussion

Results from this pilot study support feasibility and acceptability of a brief interactive weight-inclusive care (WIC) training for primary care providers (PCPs) working in a Federally Qualified Health Center. Participants actively engaged in discussion, valued clinical scenarios to reinforce key concepts, and reported confidence distinguishing WIC from weight-centric care (WCC) following the intervention. The mixed methods design provided further insight into how providers interpreted and applied WIC principles, revealing considerable variability in their ability to translate knowledge into clinical practice. Although providers readily acknowledged that health goes beyond weight, many continued to incorporate weight-centric assumptions in their clinical reasoning. This pattern, reflected in the “disconnect” theme, suggests providers may intellectually endorse WIC principles yet default to WCC when assessing a patient or formulating treatment recommendations. For example, one participant equated use of respectful, non-stigmatizing language with practicing WIC while simultaneously attributing disease processes to the patient's weight. These findings suggest adoption of WIC requires more than learning new terminology or recognizing the harms of weight stigma; it requires a fundamental shift in clinical reasoning and decision making.

Although significant changes were not detected on quantitative measures, qualitative findings suggested providers were already beginning to modify their clinical environments and workflows. Common modifications to the clinic environment included moving the scale to a more private location and increased accessibility of larger sized equipment and gowns. Reported clinical workflow changes updated policies on weighing, documentation of BMI, and asking for patient permission before discussing weight. These early changes suggest behavioral implementation may precede measurable changes on the behavioral measures used in this study and may explain the apparent discrepancy between qualitative and quantitative findings. The lack of statistical significance likely also reflects the pilot nature of the study, brief intervention, short follow-up period, and exploratory behavioral measures.

To the best of our knowledge, this study was among the first (58) to pilot a WIC training for primary care providers that explicitly targeted clinician behavior rather than attitude change. This approach is novel, as existing interventions typically focused on reducing explicit or implicit bias with modest, temporary effects (59). Further, these interventions rarely examine whether provider behaviors actually change in clinical practice (53, 60, 61). This is notable in light of growing evidence that cognitive changes are not an essential prerequisite for behavior change (62–64). Rather than attempting to modify provider attitudes before expecting practice change, this intervention drew upon behavioral principles of shaping and reinforcement to encourage adoption of WIC behaviors. The reported environmental and workflow changes may be early indicators of this process, suggesting that the reinforcement of desired clinical behaviors may provide a pragmatic strategy for de-implementation of WCC while promoting uptake of WIC practices. By shifting treatment targets toward modifiable health promoting behaviors, social determinants of health, and collaboratively developed, personalized and sustainable treatment goals (65–68), WIC offers a behavioral framework with the potential to reduce weight stigma and increase health equity.

Participants also briefly identified implementation barriers, including resource constraints, competing priorities, prior training rooted in WCC, leadership and team-related factors, and need for ongoing implementation support. Although these issues were not discussed in sufficient depth to constitute a separate emerging theme, they further illustrate the complexity of translating WIC into routine clinical practice. Similar barriers have previously been reported among providers attempting to adopt WIC, including lack of formal WIC training, organizational expectations to continue WCC practice, and reimbursement structures reinforcing traditional weight-centric models of care (65, 69). Together, these findings suggest implementation of WIC extends beyond the training, knowledge, and attitudes of individual providers and may require sustained organizational and systems-level support.

These challenges likely reflect the broader dominance of WCC throughout healthcare. Weight-centric assumptions continue to underlie medical training, clinical practice guidelines, reimbursement requirements, and healthcare infrastructure, making adoption of WIC a substantial paradigm shift (1, 38, 48, 65). For example, organizational practices may prioritize clinical guidelines that continue to use BMI to classify individual health risk, weigh all patients as a part of their appointment workflow, and provide weight loss counseling as a standard matter of practice. Providers attempting to implement WIC may thus encounter competing expectations – to reduce weight stigma and provide individualized patient-centered care while continuing to operate within systems that position body weight as the central indicator of health with weight loss as a treatment target. Although organizations such as the American Medical Association have acknowledged the limitations of BMI as a primary measure of individual health (70), their statement failed to critique the medicalization of fatness, with clinical recommendations largely continuing to operate within a WCC framework. Similarly, recent calls for systems-based and intersectional approaches to reduce weight stigma have not consistently addressed the ways in which WCC remains deeply embedded within healthcare itself (71). Implementing WIC therefore requires not only changes in individual provider practice, but reconciliation with clinical guidelines, workflows, documentation practices, training, and organizational expectations that may continue to reinforce WCC.

As a pilot study, there are several limitations to note. Training was delivered during a single provider meeting to address time constraints; only PCPs participated to maximize relevance of clinical discussions. All interview participants agreed additional training would be helpful, with specific requests for booster sessions, training other clinical staff, assistance developing individual and clinic goals, and establishing quality improvement mechanisms to promote accountability and incorporate feedback into practice changes (e.g., through virtual shadowing and monthly check-ins). Self-reported practice change was not independently verified and is more accurately framed as evidence of preliminary implementation rather than demonstrated intervention effects. Future implementation efforts may therefore benefit from delivering training across multiple shorter sessions and incorporating WIC principles into existing quality improvement initiatives to promote accountability and sustained practice change.

Additional methodological limitations should also be acknowledged. This training explicitly addressed limitations of WCC to demonstrate importance of practice change and provided supplemental resources on WIC, though utilization of these materials was not evaluated. Survey items were adapted from previous research examining weight stigma among healthcare providers for comparison purposes, with wording modified to minimize stigmatizing language (72). Included WIC behaviors were informed by ASDAH and NAAFA recommendations but were not validated prior to this study and may require further refinement to increase sensitivity to change. The 5-point Likert scale may have also limited discriminability. The small sample size, clinic and clinician characteristics, and short follow-up period also limit generalizability of findings. Finally, all data was self-reported. Surveys were anonymized to encourage honest responses, but may still reflect social desirability bias or personal blind spots. Future studies would likely benefit from additional data sources (e.g., chart review of clinical documentation, supervisor or peer observations, patient satisfaction surveys) to triangulate results. Integration of WIC tools into electronic health records, such as custom dot phrases to support weight-inclusive documentation, may further facilitate WIC adoption.

Given that adoption of WIC represents a broader shift in the conceptualization and delivery of care, our findings suggest reducing weight stigma in healthcare may require multilevel interventions and implementation strategies that extend beyond education alone. Sustainable adoption of WIC may depend on organizational leadership, supportive clinical workflows, policy and reimbursement structures, integration into professional training and clinical guidelines, and continued implementation support. Future studies should prioritize validating behavioral measures of WIC, evaluating strategies that provide ongoing implementation support to facilitate meaningful practice change, and examining longer-term outcomes.

Our findings also highlight the complexity of translating knowledge into clinical practice and suggest that paradigm shifts may remain challenging even when providers support change. An important component of this transition may be addressing the misconception that weight loss recommendations are consistent with weight-inclusive care. Researchers and clinicians should engage in clear, consistent messaging that distinguishes health from weight and emphasizes evidence demonstrating that health promoting behaviors can improve patient outcomes independent of weight change. By centering health-promoting behaviors and outcomes that directly support patient health, WIC may improve health and wellbeing while reducing the potential harms associated with weight stigma and weight centric care. Beyond consistent messaging, interventions targeting clinician behavior, when combined with structural and institutional supports, may facilitate more meaningful and sustained practice change. Future research is needed to determine which combinations of provider-, organizational-, and system-level implementation strategies are most effective in supporting the adoption and sustainability of WIC and advancing more equitable primary care.

## Data Availability

The datasets presented in this article are not readily available because participants did not consent to public sharing of data. Requests to access the datasets or training materials should be directed to Mindy McEntee, mindy.mcentee@asu.edu.

## Footnotes

1 Attendees were provided a 2022 study by Mauldin et al. (65) focusing on the harms of the weight-centric paradigm with accompanying infographic, a 2023 article by McEntee et al. (68) focusing on weight stigma within eating disorder treatment, Health At Every Size (HAES) Health Sheets (77), Eating Disorders: A Guide to Medical Care from the Academy for Eating Disorders (73), Association for Size Diversity and Health (75), Ragen Chastain's (74) Weight and Healthcare Newsletter, Gaudiani Medical Minute YouTube Series (76), a 2022 article by Vo and Golden (79) focusing on the medical complications associated with atypical anorexia nervosa, and Harvard's Implicit Association Test (78).
