# Supplementary material for: Implementation of weight-inclusive care in primary care: a mixed-methods pilot study of a brief, behavior-focused provider training
Source: Front Public Health. 2026 Sep 11;14:1934441. doi: 10.3389/fpubh.2026.1934441 (PMC13613498; doi:10.3389/fpubh.2026.1934441)
Supplement: Supplementary file 1 [file Table_1.docx]

Supplementary Material

## Supplementary File 1. Standardized patient vignette

Sofia is a 62-year-old female with an appointment at the clinic today for newly diagnosed osteoarthritis. Sofia reports pain, stiffness, and swelling in her hips, back, and knees that has gotten worse in the past few months. She was reluctant to make her appointment with you today because she was told that losing weight would fix her arthritis. She has attempted to lose weight, but has been unsuccessful. This is her first time seeing you today because she is tired of being in pain and would like to be evaluated for other treatment options.

**Vitals:** Temperature: 98.1	Pulse: 82	Blood Pressure: 129/78

**Medical History:** osteoarthritis, depression, hyperlipidemia

**Medications:** statin, osteo bi-flex (glucosamine and vitamin D), tylenol and ibuprofen PRN

**Dieting History:** Reports several attempts to lose weight during her lifetime, weight has changed between 20-110 lbs over the past 5 years. She is currently at the highest weight she has been in her lifetime.

**Nutrition:** Sofia reports she has been trying to take osteo bi-flex, but that it is expensive and she can’t always fit it into her budget. She shops at Walmart and Fry’s; grocery shops once every 2 weeks. She tries to purchase shelf stable foods so that she doesn’t end up with a lot of food waste. When preparing meals at home, she tries to stick with things that are quick and easy. She reports it hurts to stand for long periods of time to cook. She feels hungry most of the day and tries not to eat snacks (in effort to lose weight). She waits ~5 hours between each meal to eat.

**Movement:** Sofia reports it is hard for her to go up and down stairs and to walk distances. She feels stiff when she wakes up in the morning and it hurts to exercise. She doesn’t enjoy “exercise.” She likes swimming and has access to a community pool, but doesn’t like wearing a bathing suit because she is concerned about what others think of her body. She has attended a couple of Silver Sneakers classes but they are usually in the morning when she is working. She is having difficulty with her day-to-day activities because of the pain - especially grocery shopping and keeping her house clean.

**Social History:** Sofia is a veteran who served in the Navy for 8 years as a Search and Rescue Medical Technician when she was in her 20’s. She currently lives in Yuma, AZ. She receives $1,000 per month for disability from the VA. She works from home as a medical scheduler for General Surgery. Combined with her VA benefits and current job, she earns $46,000 per year. She has health insurance through her job and access to the VA for medical care. She lives in a 55+ community. She has a car but is afraid to drive it because it is old and in need of a lot of repairs. She is nearing retirement, but worried about having enough money to support her during retirement.

“Things have gotten harder since my body size has increased. My clothes don’t fit right and are uncomfortable. I put off seeing my friends because I am ashamed of my size. I don’t fit into chairs easily anymore.”

## Figures

|  | Undecided | Agree | Strongly Agree |
| --- | --- | --- | --- |
| Collaborate on non-weight treatment goals |  | 8 | 2 |
| Communicate respectfully about health and weight |  | 9 | 1 |
| Distinguish weight-centric and weight-inclusive care | 1 | 8 | 1 |

**Supplementary Figure 1.** Self-Rated Weight-Inclusive Care Skills Post Intervention (n=10)
